# Care and management of a double burden of chronic diseases: Experiences of patients and perceptions of their healthcare providers

**DOI:** 10.1371/journal.pone.0235710

**Published:** 2020-07-16

**Authors:** Nasheeta Peer, Anniza de Villiers, Deborah Jonathan, Cathy Kalombo, Andre-Pascal Kengne

**Affiliations:** 1 Non-communicable Diseases Research Unit, South African Medical Research Council, Durban and Cape Town, South Africa; 2 Department of Medicine, University of Cape Town, Cape Town, South Africa; 3 Metro-District Health Services, Gugulethu, Cape Town, South Africa; University of the Witwatersrand, SOUTH AFRICA

## Abstract

**Aim:**

The increasing burden of comorbid HIV infection and hypertension necessitates a focus on healthcare services providing care for chronic multi-morbidities. The aim of this study was to evaluate the perceptions and experiences of 1) people living with HIV infection and comorbid hypertension, and 2) their healthcare providers, related to their diagnoses and interactions with chronic healthcare services in South Africa.

**Methods:**

This study comprised quantitative and qualitative arms with a multi-layered approach. We randomly selected 17 public healthcare facilities providing HIV care across Cape Town and surrounding rural municipalities.

**Results:**

Interviews were conducted with clinicians (n = 11), specialised nursing professionals (n = 10), lay counsellors (n = 12), six patients focus groups (n = 35) and 20 in-depth individual patient interviews. There were mixed views on being treated at integrated vs. separate chronic care facilities regarding quality of care and privacy/anonymity. Specialised clinics offered better care for HIV infection while hypertension and other non-communicable diseases were neglected. Privacy about HIV status maybe better maintained in integrated clinics but not if status was disclosed by having the green-coloured HIV treatment card. A single appointment date was considered advantageous as it saved time and money leading to greater compliance; however, waiting times at clinics were longer with perhaps fewer patients seen.

**Conclusions:**

The mixed reactions elicited to the integration of healthcare services for HIV, hypertension and other non-communicable diseases highlights the complexities involved in implementing such services. Greater human resources with retraining and reskilling of healthcare staff is required for the optimal management of chronic multi-morbidities.

## Introduction

South Africa’s healthcare services are struggling to cope with the quadruple burden of disease, comprising human immunodeficiency virus (HIV) infection, cardiovascular and other non-communicable diseases (NCDs), poverty-related diseases, and violence and injuries [1, 2]. The country has the greatest burden of HIV infection globally; almost eight million adults in South Africa were HIV positive in 2019 [3] with one in five (19%) South Africans aged ≥15 years being HIV positive [4]. Additionally, hypertension, a major NCD risk factor in both the HIV infected and uninfected populations, is prevalent in about 45% of South Africans [4]. This overall hypertension prevalence is comparable to the 46–48% hypertension prevalence reported in HIV-infected adults in a community-based study conducted in Agincourt in rural South Africa [5] but higher than the 22% reported in HIV positive antiretroviral (ARV) naïve patients from a study conducted in eight public sector clinics across South Africa [6]. The significant burden of hypertension and other NCDs in people living with HIV (PLWHIV) is ascribed to their increasing longevity and exposure to NCDs risk factors.

The focus of care is currently on infectious diseases, particularly HIV infection and tuberculosis, and overburdened healthcare services and health professionals in South Africa generally perceive NCDs as less urgent [7]. However, effective combination ARV therapy has resulted in the overall mortality associated with HIV infection decreasing significantly and life expectancy increasing to the extent that NCD-related deaths now represent a rising proportion of mortality in HIV-infected South Africans [8].

Considering that the risk of NCDs has emerged as an important consideration in HIV-infected individuals and is likely to increase as they live longer [9], greater attention needs to be paid to the care of PLWHIV and NCD comorbidities. Seeing that the requirements for effective decentralised provision of therapy for HIV infection and for NCDs are closely aligned [10], the South African National Department of Health initiated the Integrated Chronic Disease Management Model in 2011 [11, 12]. This model incorporates a diagonal approach that integrates the vertical HIV programme with the horizontal general healthcare system [13, 14].

In order to appropriately allocate resources and develop cost-effective therapeutic strategies and programmes for the integrated and simultaneous care of comorbid conditions like HIV infection and hypertension and other NCDs, there is a need to determine the efficiency, quality and acceptability to users, and the effects on health status of integrating care for chronic infections and NCDs [10, 15, 16]. Therefore, the aim of this study was to evaluate the perceptions and experiences of PLWHIV and hypertension comorbidity on hypertension diagnosis, management and integration within HIV care, and the healthcare providers who deliver such services at ARV clinics in the Western Cape Province of South Africa. This will provide ‘on the ground’ insights i.e. through their own voices from individuals who both utilise and provide such services, which will enable healthcare services and systems to improve delivery of integrated healthcare services.

## Methods

### Study design and participant selection

**Fig 1:**
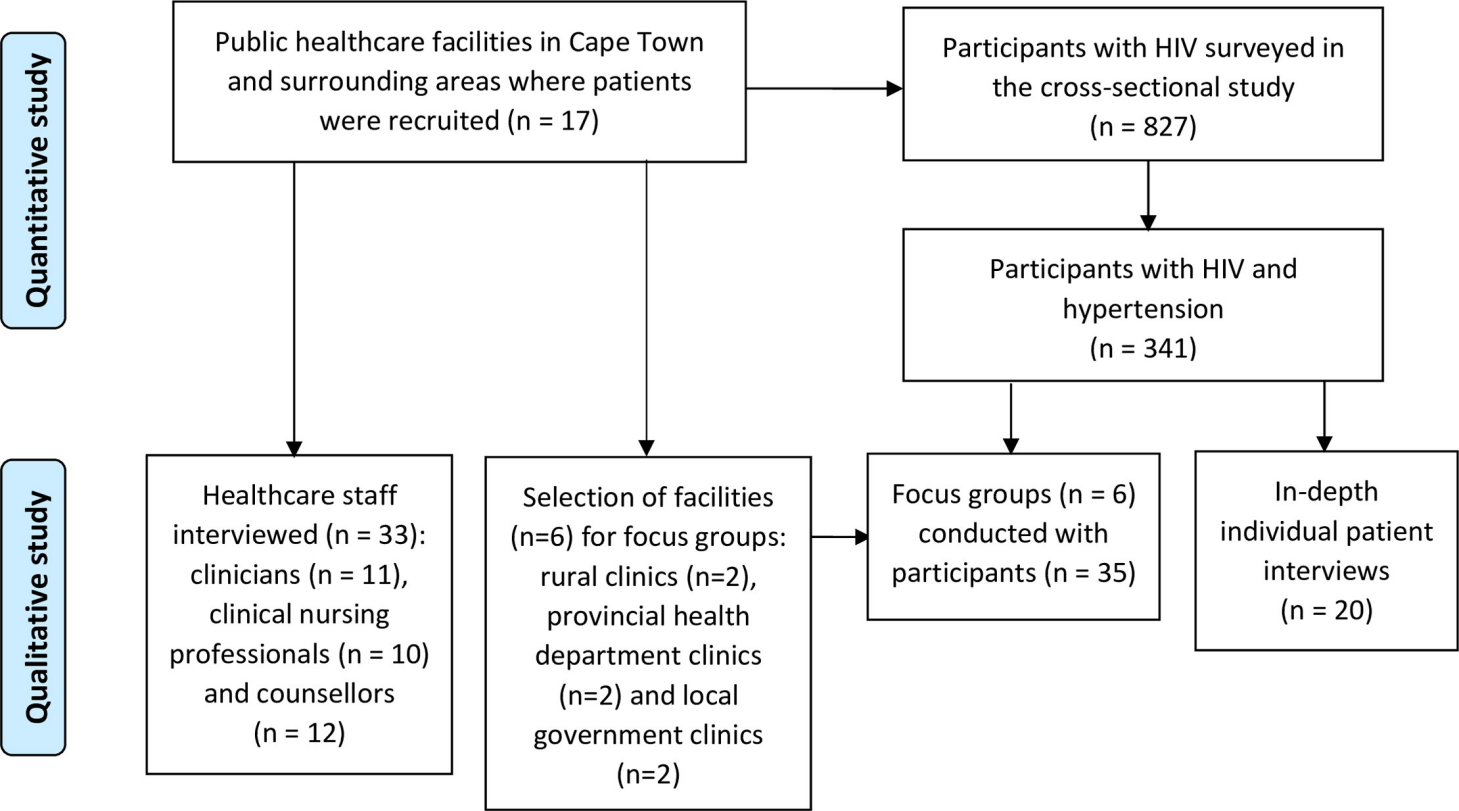
Sampling of study participants.

This study comprised both quantitative and qualitative arms, with the latter the focus of this paper (Fig 1). The cross-sectional survey, conducted between March 2014 and February 2015, was followed by the qualitative data collection, which was completed between March and May 2015. Briefly, 17 public healthcare facilities in Cape Town and the surrounding rural municipalities were randomly selected from a list of facilities that treated more than 300 HIV infected patients monthly, for the quantitative arm of the study, and has been described in detail previously [17].

For the qualitative arm of this study, a multi-layered approach comprising three interview phases was utilised. Firstly, interviews, guided by semi-structured schedules were conducted with staff at these healthcare facilities by two investigators experienced with in-depth interviewing. Staff interviewed included those who 1) were in direct contact with patients i.e. counsellors, nursing professionals and clinicians, 2) had been in their positions for more than three months and 3) availed themselves for interviews on the days that the facilities were visited.

Secondly, focus groups were conducted with HIV positive participants who had hypertension and who were on both ARVs and anti-hypertensive medication. These participants were conveniently selected from survey participants (i.e. those who had completed the quantitative arm of this study) at six facilities (Fig 1). These facilities were purposively selected from the 17 facilities where the quantitative arm was conducted to allow for an equal distribution of two facilities each to be included from the rural, provincial health department and local government clinics. This provided an opportunity to engage with patients accessing different public healthcare services.

Thirdly, participants diagnosed with hypertension before or during the survey were randomly selected from the securely stored participant database, contacted, and invited for in-depth interviews (Fig 1). None of these selected participants had participated in the focus groups. A single experienced investigator, assisted by an experienced translator, conducted these interviews.

### Data collection and analysis

The interview and focus group schedules were developed using insights obtained during the completion of the survey i.e. the quantitative arm of the study. These aimed to answer some of the broader questions posed by the research i.e. perceptions and attitudes of 1) HIV-clinic attendees and 2) healthcare providers to co-management of HIV and NCDs.

This study utilised and adapted the “framework for understanding diabetes care within the context of comorbid chronic conditions” as described by Piette and Kerr [18]. The aim of this framework is to aid health systems and researchers in improving care in a setting of comorbidities. Importantly, this model enables the understanding of why specific health system changes sometimes have little impact on patients’ health status and their utilisation of healthcare services.

The interviews and focus groups were recorded, professionally translated, transcribed and analysed utilising ATLASti software and applying the six steps of thematic analysis [19]. Scientists familiar with the local HIV treatment milieu analysed and interpreted the data. The same research team conducted the interviews, which was deemed appropriate, because they were not ‘insiders’ and had no preconceived ideas or vested interests in the responses and perceptions of the participants. Analysis was done by the co-author (AdV); the themes were predetermined by the interview schedule and these broad themes were then scrutinized for subthemes. The broad themes were then revisited and, if necessary, renamed. Although no inter-rater reliability was established the analysis included a consensus seeking process between a small sub-group of the research team. Expert checking of selected themes, codes and quotations was done by a seasoned clinician (CK) attached to a large HIV treatment facility. The final step of the analysis process aimed to provide a narrative report of the perceptions and experiences of the participants deemed most salient by team members. Although this could constitute bias and allow for the researchers to influence what is presented, the connection the research team had with the experiences of HIV positive individuals after a year of collecting data and interacting extensively with the participants in the survey phase of the study provided opportunity for ensuring that the analysis process delivered a valid and reliable report.

### Ethics approval and consent to participate

All procedures were performed in accordance with the ethical standards of the South African Medical Research Council and conducted in accordance with the principles of the Declaration of Helsinki of 2013. Approval was granted with the protocol ID EC021-11/2013. Permission to conduct the survey was obtained from Health Research Office of the Western Cape Department of Health, and the relevant healthcare facilities. Informed consent was obtained from all individual participants included in the study.

## Results

**Fig 2:**
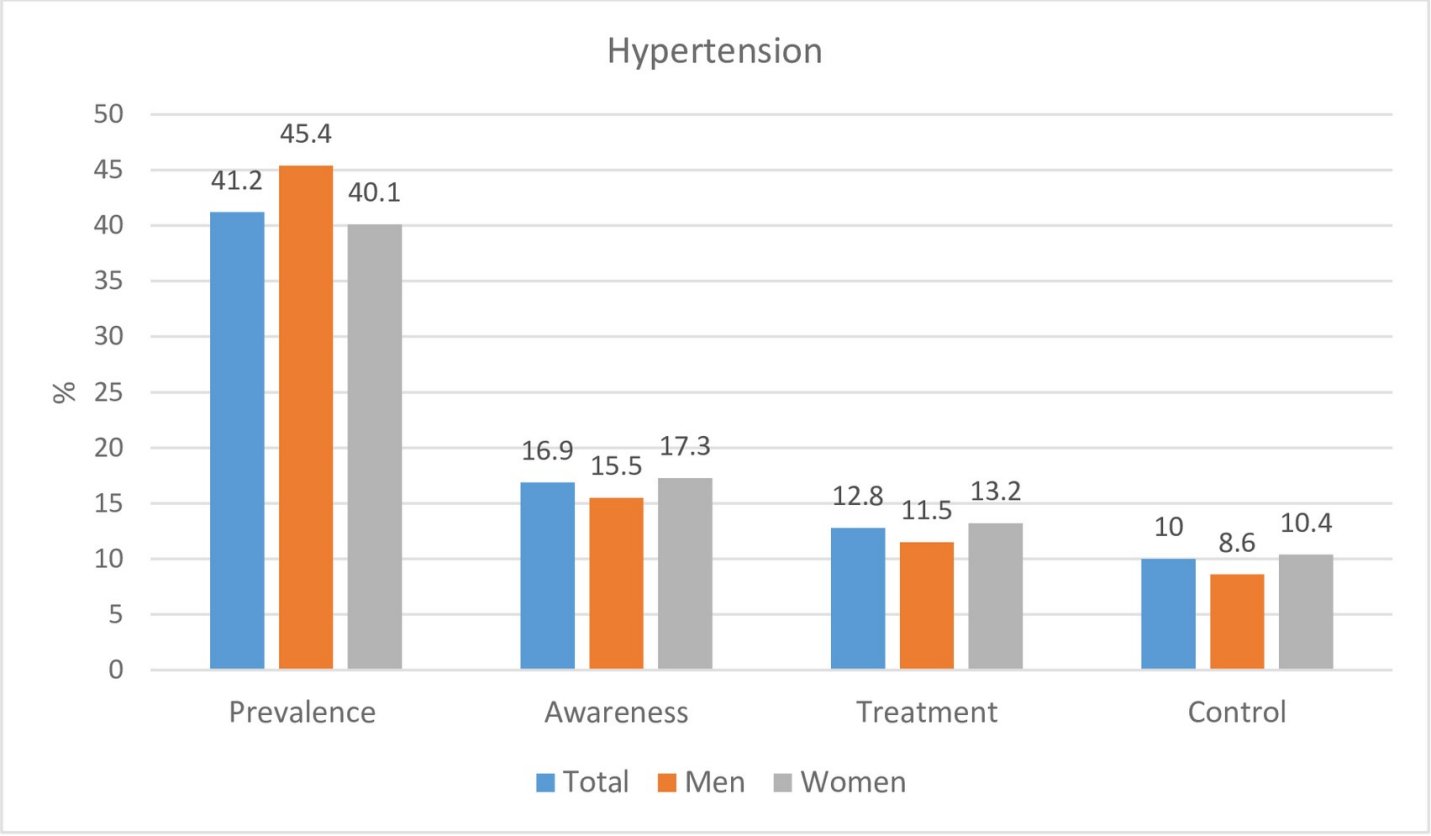
Prevalence of hypertension in the overall sample (n = 827), and hypertension awareness, treatment and control among participants with hypertension (n = 341).

For context, among the 827 HIV-infected participants attending ARV clinics in Cape Town and who were included in the quantitative arm of this study, 41.2% had hypertension (Fig 2), defined as BP ≥140/90 mmHg or the use of antihypertensive agents [20]. Among those with hypertension, awareness, treatment and control of their hypertension was low at 16.9%, 12.8% and 10.0%, respectively. This demonstrates sub-optimal management of a highly treatable and serious condition in patients regularly attending healthcare services.

In the qualitative phase of this study, 33 healthcare professionals (11 clinicians, 10 clinical nursing professionals and 12 counsellors), and 55 patients (35 in six focus groups and 20 in-depth individual patient interviews) were interviewed. These participants had been diagnosed with HIV infection and with hypertension for an average of 7.5 years and 6.5 years, respectively. Two participants were diagnosed with hypertension during the survey but had since been taken off medication. Six participants with hypertension also had diabetes; although the focus of this study was on hypertension, participants’ experiences with diabetes have been included because of the common co-occurrence of these two conditions. Participants considered their health to be fair but about 25% did not know the values for normal or high blood pressure.

Two themes were explored in this paper. The first theme describes living with comorbid HIV infection and hypertension 1) as experienced by PLWHIV and 2) as perceived by healthcare providers. The second theme explores the interactions with the primary healthcare (PHC) system 1) as experienced by participants with comorbid HIV infection (PLWHIV) and hypertension, and 2) as perceived by healthcare providers of their patients’ experiences. The key findings are presented in Table 1.

**Table 1:** Using a framework for understanding care within the context of comorbid chronic conditions to explore the experiences of people living with HIV (PLWHIV) infection and hypertension, and their healthcare providers perceptions.

	Sub-themes	Patients’ experiences	Healthcare providers’ perceptions
Patient resources and priorities for HIV management	Ability to cope	• Stigma attached to having the “green card” for antiretroviral (ARV) therapy, which identifies being HIV positive.	• Having an HIV diagnosis is difficult for patients
Clinical resources and priorities for HIV management	Healthcare system	• Long waiting times at clinics. • Treated unfairly when they reschedule appointments by being attended to after all other patients • Attended to by different doctors at each visit with lack of continuity of care. • Holistic management with other problems also being attended to i.e. hypertension diagnosed at the HIV clinic.	• Specialised quality care provided for PLWHIV
Patient resources and priorities for comorbid non-communicable diseases (NCDs) (hypertension and/or diabetes) management	Ability to cope	• Hypertension is easier to have than HIV • Acceptance of NCD diagnosis because of diagnoses among family members and expect to develop the same conditions • Acceptance of NCD diagnosis because already had HIV infection • Hypertension diagnosis more painful because told by HIV counsellor previously that HIV not as dangerous as hypertension • 25% did not know the values for normal or high blood pressure	• Hypertension is fine, but diabetes is more difficult to control than HIV • Maybe more accepting of an NCD diagnosis than HIV • Resigned to an NCD diagnosis because of a family history • Complaints about medication i.e. a high pill burden • NCD diagnosis has less stigma than an HIV diagnosis • Stressful to be diagnosed with more than one chronic condition • Fearful of NCD consequences such as stroke and mortality • Coping with health-related consequences of NCDs is difficult
Clinical resources and priorities for comorbid NCDs (hypertension and/or diabetes) management	Healthcare system	• NCDs do not receive the same level of care as HIV infection • Providing integrated care through the general system will likely remove the stigma attached to the ARV clinic • Integrated care may lead to greater treatment seeking behaviour and fewer defaults • Integrated care may lead to longer waiting times and less personalised care	• Accessing treatment for HIV and NCDs at different clinics is difficult • Needing to attend multiple clinics leads to missed appointments • Integration of NCD and HIV services are advantageous for patients as it saves them time and money • Integration of NCD care in ARV clinics will lead to better management • Better care offered by a single vs. multiple healthcare provider • Integration of services leads to longer waiting times • Anonymity re HIV status may be better maintained in integrated clinics • Some patients prefer different clinics to hide their HIV status because of stigma attached to HIV infection Challenging for younger HIV-infected patients to have NCDs and be treated alongside people their parents’ age • Integration of care would lead to less specialised care for PLWHIV

### Theme 1: Experiences of comorbid HIV and hypertension diagnoses

#### PLWHIV

Participants shared their experiences with the researcher of when they were first diagnosed with hypertension and their subsequent management of the two morbidities. More than half of the interviewed participants indicated that they accepted the hypertension diagnosis despite the initial shock.

Initial shock but positive attitude: *“It was shocking news and I tell myself it will not kill*, *but I will kill the high blood” (Site F)*

Having a family history of hypertension and being already diagnosed with HIV infection played a role in making the diagnosis less distressing.

Easy acceptance because of HIV status: *“It was okay because I was diagnosed with HIV already” (Individual Interview*, *Site E)*

Easy acceptance because of family history: *“I didn't find so difficult cause members of family they are on medication for hypertension and I told myself it's a family thing” (Individual Interview*, *Site A)*

However, other participants described negative emotions upon being informed of a hypertension diagnosis or associated with having multi-morbidity. These included having additional stress and feelings of hopelessness, and a fear of dying.

Blood pressure affected by stress of daily living and feelings of hopelessness: *“Because of stress of being unemployed my high blood is always high*. *When you are on these medication like you taking two different medication and do grant you don’t get it*. *You go and look for a job you don’t get it*. *And I am not educated*, *there are jobs in other places*, *but I don’t qualify*.*” (Focus Group*, *Site F)*

Fear of death: *“It was more painful*, *because the counsellor who spoke to me when I was diagnosed with HIV said it is not dangerous like high blood pressure and diabetes*, *these ones they will kill you*.*” (Individual Interview*, *Site E)*

#### Healthcare providers

Most of the quotations on healthcare provider perceptions of their HIV positive patients’ experiences with an NCD co-morbidity were generated from the counsellors, followed by the nursing professionals and the clinicians. The strongest sub-theme emerging across all three staff categories was that living with an NCD-related morbidity was distressing for their HIV positive patients. A high pill burden and coping with health-related consequences of the conditions emerged as factors that may be associated with this experience.

Pill burden: “*They feel bad because they say there is a lot of medication they must take*, *there is a lot of them that they must take*, *both ARVs and maybe diabetic or hypertension*, *they must take both of them at the same time*, *so they complain a lot about a lot of medication” (Counsellor*, *Site H)*

Immediate health-related consequences: “*They are free but in this sugar diabetes and hypertension they feel that now they are stressed because they know that each and every time they’ve had to go to the clinic or sometimes they got sick …they might get diarrhoea and everything whenever that it goes it means more medication*, *more medication*, *more medication than for the HIV” (Counsellor*, *Site A)*

Awareness of long-term disease related consequences: *It’s difficult for them to handle the HIV positive status and now you are starting now to diagnose another chronic disease of which they know the consequences of the strokes*. *Maybe they have a family member that died that had hypertension before” (Clinical Nursing Professional*, *Site B)*

According to healthcare providers, PLWHIV believe that it is more difficult to control diabetes, but not hypertension, compared with HIV infection because they see their family members suffering from diabetes.

Diabetes: *“I am hearing it more and more*. *If I say to them unfortunately I have to tell you today you have got sugar disease and then they tell you*, *… how did that happen*. *I should have just stayed with my HIV because they’ve got this thing they’ve see their mothers and family members come through the diabetes they feel HIV is better controlled than diabetes*. *Hypertension is fine*.*” (Clinical Nursing Professional*, *Site L)*

Healthcare providers reported that younger PLWHIV perceive that NCDs are generally associated with older age, so they now have conditions associated with people of their parents’ age.

Life stage: *“For the younger ones it’s a bit more of a challenge because*, *you know*, *you’re 35 years old*, *you’ve got HIV*, *it’s a lot to carry already*. *It’s a lot of burden in terms of the stigma*, *and now you’ve got hypertension also*. *And I think those are the challenge*, *because now that 35-year-old must go and sit in another club with people who could be your parents…*. *So there’s also that sort of stigma*, *you know*, *I’m 35-year old*, *I’m young and could be my mother*, *my father now sitting here*.*” (Clinician*, *Site B)*

On the other hand, some healthcare providers also reported that there was no stigma attached to a diagnosis of hypertension or diabetes, and that patients accepted the diagnosis and paid the same level of attention to their NCDs as they did with their HIV care.

No stigma: *“Well I think they are probably much more acceptant of having hypertension and diabetes rather than having HIV*. *A lot of them actually lie already to their bosses and say that they are coming to the clinic for high blood pressure treatment instead of coming for their ARVs*. *So they are more likely to disclose it and accept it as a diagnosis you know*.*” (Doctor*, *Site G)*

Family history: *“And you know some of them will tell you that I am expecting it within my family so there’s nothing …” (Doctor*, *Site A)*

Easier to deal with: *“…much easier to have high blood pressure than HIV” (translated*, *Clinical Nursing Professional*, *Site Q)*

In summary, there was no stigma attached to a diagnosis of hypertension compared with HIV, and some participants were accepting of the hypertension diagnosis because of having a family history of hypertension or already a diagnosis of HIV infection. However, some felt that NCDs particularly diabetes, were more difficult to control than HIV because of the experiences of their family members. Further, some younger participants linked NCDs with older age and felt they had conditions associated with their parents’ generation. Many participants described hypertension as an additional burden in their already difficult lives; it was associated with greater distress because of a higher pill burden and coping with the health-related consequences.

### Theme 2: Experiences with the PHC system

#### PLWHIV

Participants’ experiences, elicited from the focus group discussions, described their interactions with the healthcare system and provided some insight on how they perceived these interactions, including receiving integrated healthcare. The services provided for NCDs varied across the six facilities where the focus group discussions were conducted. These ranged from fully integrated care provided at the ARV clinic for both HIV and NCDs, to certain services for NCD related care, to only HIV care. In the latter instances, care for NCD related co-morbidities had to be accessed at a different site which may have been in the same healthcare centre or at a facility some distance away. As these levels of care were not included in the study design the findings do not attempt to compare or distinguish between sites.

More codes were generated pointing to negative rather than positive experiences with the healthcare system. While several codes supported negative experiences with doctors these were all generated from a single focus group that included participants from two ARV centres. Many participants were unhappy with the lack of continuity of care received. They were seen by different doctors who diagnosed them with different conditions each time they visited the clinic. Moreover, the doctors were inattentive and did not provide clear explanations for their diagnoses nor the investigations that were undertaken. This seemed to have been a source of anxiety and uncertainty.

Clinic staff: *“If it was for me I want to be seen by one doctor*, *reason being in the past years ever since I started attending the clinic after diagnoses I’m being seen by different doctors*. *This doctor will say you have diabetic and on another appointment I see another doctor and he will say you have high blood*, *next appointment I see another doctor*. *Why they say we must draw bloods for INR*? *What is INR I became stupid*, *for me it’s the first time I feel okay this year ever since I met the new doctor that I see” (Focus Group*, *Site B)*

Long waiting times at the healthcare centres, specifically in pharmacy lines, generated discussion in all focus groups. Patients arrived very early in the mornings but left only in the afternoon; the major part of their day was spent at the clinic waiting in different queues.

Long wait: “*I go home at home at 14h00*. *It takes me from 06h00 to 14h00*. *It is a lot*.*” (Focus Group*, *Site J)*

Other experiences perceived as unfair by patients related to clinic procedures, such as poor treatment during a rescheduled appointment. Participants were unhappy about being discriminated against when attending a rescheduled appointment. These appointments were not adhered to by the clinic staff who made them wait a few hours longer or till all other patients had been attended to. Participants felt that this was unreasonable considering that the rescheduled appointment had been agreed upon.

Clinic procedures: *“And then sometimes if maybe you miss your appointment maybe your appointment was yesterday and then they said you must call*, *and then you call then to make an appointment maybe for today but when you come maybe at 8 o’clock or 7 o’clock or 9 o’clock the receptionist they will say you must wait until 11 or until everybody is finished*, *after they are finished with those with appointments they will help you*. *It’s really funny because you called them and you told them that maybe you’ve got a problem you were supposed to come and they said you can come tomorrow then why they let you sit there*?*” (Focus Group*, *Site F)*

Some participants also mentioned their fear of being stigmatised for having HIV infection. This was related to owning the green-coloured treatment card which signified being HIV positive. People were thereby made aware of their HIV status and they were stigmatised prior to attending the ARV clinics.

Stigma attached to the green ARV card: “*My problem is really the green card*, *once you take out the green card they know where you are going*, *the stigma starts from the card not from the clinic” (Focus Group*, *Site N)*

A few participants specifically reported that the system provided less care for their NCD co-morbidity than for their HIV condition.

Less care in the system for NCDs: *“Do you know many times they tell me*: *Sister I have taken my folder for the chronic medication but you know the diabetic and hypertensive they do not get the same care*.*” (translated*, *Clinical Nursing Professional*, *Site Q)*

Participants, however, also reported positive experiences with the healthcare system. Some participants spoke highly about their doctors who provided holistic care. For example, a complaint about headaches was investigated further and a diagnosis of severe hypertension made, thereby averting a potential stroke.

Integrated care: *“I used to collapse almost every day*. *I could not speak I couldn’t open my eyes*. *I could say I almost had a stroke*, *but luckily*, *I could say thanks to God because it didn’t go that far*. *For the clinic (ARV) I could say that they are very helpful*. *Because even now when doctors picked up that I had high blood*. *Every time when I’m coming to the doctor I’m complaining about the headaches and sometimes I don’t sleep at night and the doctor said it can’t be only the depression*, *let us check what could be maybe it’s something else*. *Then they started to check for high blood and sugar*, *and my BP was too high it was like 300 and something*, *and then they started to put me on treatment of high blood*.*” (Site F)*

Participants pointed out that providing integrated care through the general system will likely remove the stigma attached to the ARV clinic, which may lead to greater treatment seeking behaviour and fewer defaults. However, there was likely to be longer waiting times and less personalised care.

#### Healthcare providers

Healthcare providers’ insights into how PLWHIV may or do experience receiving integrated care for their HIV infection and NCD related co-morbidities complemented those of their patients. Some patient perceptions were confirmed by healthcare providers who worked in healthcare centres where integration of HIV and NCD services had already been implemented. Integration of care in the ARV clinics was identified as the best treatment scenario by some participating clinicians.

Better care: *“Obviously for the patient it is better to receive all your care from well one provider*, *it makes a lot more sense*, *it is better care for the patient and the patient only needs to come fetch medications once” (Clinician*, *Site J)*

However, others felt that this would lead to less specialised care for PLWHIV and to far fewer patients being seen daily if no additional staff was provided.

Longer waiting times: *“… a*, *since the integration*, *I don’t know if it’s short of staff or what*, *but they stay longer that they expect*, *so they complain now*, *about the times” (Counsellor*, *site H)*

Other perceived positive outcomes related to the integration of healthcare services was that it would be advantageous for patients as it would save them time and money. This could potentially lead to fewer missed clinic appointment dates, which arose from having to access treatment at multiple sites.

Cost and time benefit: *“It saves money for the patient–look lots of the patients are working*, *so it saves money for them to come twice to the facility” (Facility Manager*, *Site N)*

Burden of having to access treatments at different sites: “*And then because sometimes they don’t want to go this side and this side*, *sometimes they default to come here says I am sick and tired to go there and there*, *sometimes their appointments is the same date” (Counsellor*, *Site J)*

Furthermore, anonymity may be maintained for HIV infected patients who receive care at integrated clinics. This is important because of the stigma attached to being labelled HIV positive.

Less stigma: *“Well*, *it would be better because no-one*…*it’s a good dream*, *because there’s no-one that would feel like*, *“oh*, *that one is talking about me or is pointing me”*, *you know*. *that one is talking about me or is pointing me”*, *you know*. *To the patients it’s still the stigma of being HIV positive*, *more than high blood and diabetic” (Counsellor*, *Site B)*

However, some healthcare providers believed that integrated services will have the opposite effect i.e. patients will feel that their HIV status will be revealed.

Stigma: *“Patients are not ready for integration because they go to clinic X for ARVs and to clinic Z for other medications because they do not want to people to know about their HIV status (translated*, *Clinical Nursing Professional Site Q)*.*”*

In summary, a common key complaint was the long waiting times with the better part of the day spent waiting in queues at clinics; patients came early in the morning and only left in the afternoon. The waiting times were prolonged when they rescheduled an appointment because they were discriminated against and only attended to once everyone else was seen.

Providing integrated care through the general system was considered likely to destigmatise HIV treatment because of greater anonymity; this may lead to greater treatment uptake and fewer defaults. However, depending on how services were integrated, there were fears about HIV status being revealed e.g. when the green ARV card was displayed. Further benefits to integrated care was that patients would save on transportation costs and on time needed to access multiple clinics. Nevertheless, there were concerns around quality of care being compromised and on longer waiting times with fewer patients being attended to daily if additional staff were not allocated.

The care provided across healthcare facilities was not consistent. At some facilities doctors used a patient-centered approach, provided holistic care and communicated well with their patients, which led to hypertension being diagnosed and this was appreciated by patients. In contrast, at other clinics, patients were seen by different doctors at each visit which led to a lack of continuity of care and different diagnoses at each visit. These doctors were inattentive and lacked communication skills, which led to anxiety and uncertainty among the patients. The management of HIV was considered to be better than that for hypertension and other NCDs.

## Discussion

This qualitative study explored the experiences of PLWHIV and the perceptions of their healthcare providers of living with the comorbidities of HIV infection and hypertension, and their interactions with the healthcare system. The findings of this study describe mixed experiences and perceptions that need to be holistically considered and addressed to improve management of comorbid HIV infection and hypertension.

Although there was no stigma attached to a diagnosis of hypertension compared with HIV, many described it as an additional burden which caused distress. PLWHIV and co-morbid hypertension or other NCDs may likely require additional counselling and social support to improve their coping skills and ensure that they adhere to their treatment regimens and healthy lifestyle behaviours.

Among the key findings is that while integration of healthcare services for HIV infection and NCDs is advantageous for patients as it saves them time and money, and that better care may be offered by a single vs. multiple healthcare providers, integrated services cannot be achieved without greater investment in terms of resources. While specialised quality care is provided for PLWHIV at the ARV clinics, integration of services may lead to poorer care overall. There may be less specialised care for HIV infection or NCDs may not receive the same level of care as HIV infection. Additionally, integration of services may lead to longer waiting times was another concern.

These are valid concerns that highlight the complexities involved when integrating care for multiple chronic conditions. For the successful management of chronic multi-morbidities in a setting of modest resources, a well-organised and focused strategy by healthcare systems, in a supportive political milieu, is required to achieve health service integration with optimal outcomes [21]. Changes are required in the healthcare system at the levels of policy, organisation, training and facilities. The major challenges for the delivery of integrated care are the shortage of healthcare providers with the appropriate training and skills, and an already overburdened workforce [21], as evident in South Africa from this and other studies [11, 12, 22, 23].

Insufficient healthcare providers contributed to the long waiting times, which was a common occurrence in this study despite appointments being scheduled. Additionally, when appointments were rescheduled, participants felt their waiting times were extended even further and they were discriminated against. This accorded with a study conducted in rural South Africa where patients perceived that they were punished for missing clinic appointments and made to wait longer at subsequent visits until nurses had attended to scheduled patients [11]. Long waiting times are a barrier to optimal patient care and makes regular clinic attendance and retention of patients in care extremely difficult. This in turn affects adherence to ARV and antihypertensive medications and leads to inadequate viral load suppression and poor blood pressure control.

Greater human resources need to be allocated to cater for the greater load of patients with comorbidities; considerations need to be given to upskilling of staff for the management of comorbidities to ensure optimal care, and to providing more staff because of likely longer consultation times for patients with comorbidities. For efficient and effective chronic care delivery, specialised training in integrated chronic care management and dedicated chronic care teams are required [22]. These concerns were raised in this study where participants felt that integration of services may lead to less specialised care for PLWHIV and fewer patients attended to daily if additional staff were not provided. Such measures are, therefore, crucial to enable the successful integration of NCD and HIV services. They are also likely to allay the apprehensions of patients with comorbidities and their healthcare providers who are concerned about long waiting times, greater defaults on scheduled appointments and lower quality of care.

Additional to strengthening the PHC system for management of comorbid HIV infection and NCDs, is to address the specific fears articulated by PLWHIV. One of the concerns in this study was the importance of maintaining HIV status anonymity to avoid stigmatisation. Addressing such concerns is key to ensure that PLWHIV are comfortable with attending PHC services to optimise their care and remain healthy. This was reiterated in the rural South African study where all patients with chronic diseases were managed in the same clinics with no separation of patients or consultation rooms [11]. Patients reported greater satisfaction and their CD4 counts and blood pressures were better controlled [12]. Integrated services thereby increased anonymity, reduced stigma and encourage their utilisation. This accords with the review by Duffy and colleagues; they suggest that the simultaneous introduction of integrated HIV and NCD services or integrating HIV care into that of NCDs rather than vice versa may perhaps reduce HIV stigma on account of such services being located outside of HIV facilities [24].

However, while integrated healthcare services may allow greater anonymity, this would be nullified if PLWHIV were seen carrying the green treatment card, used for ARV treatment in the Western Cape only. In such instances, PLWHIV are likely to prefer separation of services for ARVs and NCDs. Such seemingly minor, but critical, factors that have a profound impact on the lives of PLWHIV, an extremely vulnerable population, need to be urgently addressed.

Other factors include the sensitivity with which a diagnosis of an NCD or HIV status is conveyed; it must be emphasised that one should not be compared with the other in an attempt to soften the blow of being diagnosed with HIV infection. It is likely that a significant number of patients with one diagnosis may develop the other conditions, and there is a need to ensure that they are not traumatised by the comorbid diagnoses. This requires retraining and reskilling of healthcare staff, as discussed above.

## Strength and limitations

The framework used for understanding care within the context of comorbid chronic conditions in this study ensured that patient and provider experiences and perceptions were explored utilising multiple dimensions. Another strength of the study is that we investigated the perceptions of different healthcare service providers as well as patients. Other strengths are the number and diverse range of healthcare facilities, and the different levels of integrated care provided at the facilities included in this study. This is, however, also a limitation as most of these facilities differed in the type and manner health services were offered. It is therefore not possible to link patient or provider experiences and perceptions to service type or level of integration of HIV and NCD care. About a third of staff, who may have provided additional insights, did not meet the inclusion criteria or were unavailable for interviews due to unforeseen circumstances, which is a limitation of this study.

## Conclusion

This study underscores the intricacies and difficulties associated with the diagnosis and management of co-morbid HIV infection and hypertension. Addressing the problems highlighted in this study by improving patient experiences and interactions with healthcare services will encourage their regular utilisation and potentially lead to improved care, particularly for hypertension which was unacceptably poor in this study. The mixed reactions elicited to the integration of healthcare services for HIV and hypertension and other NCDs further highlights the complexities involved in implementing such services. There is an expanding demand for chronic care of comorbid HIV infection and hypertension and other NCDs which needs to be urgently addressed. Investing in intervention measures to ensure the provision of optimal PHC integrated service for HIV infection and NCDs will have the maximum impact health wise and economically [25]. Ongoing surveillance and reviews of such services are essential to ensure their success and widespread uptake.
